# Psychophysiology and Imaging of Visual Cortical Functions in the Blind: A Review

**DOI:** 10.3233/BEN-2008-0217

**Published:** 2009-07-29

**Authors:** Stephanie L. Simon-Dack, P. Dennis Rodriguez, Wolfgang A. Teder-Sälejärvi

**Affiliations:** ^1^North Dakota State UniversityFaigoNDUSA; ^2^Indiana University South BendSouth BendINUSA

**Keywords:** Visual function, plasticity, blind, brain imaging, EEG, ERP, fMRI, PET, TMS

## Abstract

Imaging, transcranial magnetic stimulation, and psychophysiological recordings of the congenitally blind have confirmed functional activation of the visual cortex but have not extensively explained the functional significance of these activation patterns in detail. This review systematically examines research on the role of the visual cortex in processing spatial and non-visual information, highlighting research on individuals with early and late onset blindness. Here, we concentrate on the methods utilized in studying visual cortical activation in early blind participants, including positron emissions tomography (PET), functional magnetic resonance imaging (fMRI), transcranial magnetic stimulation (TMS), and electrophysiological data, specifically event-related potentials (ERPs). This paper summarizes and discusses findings of these studies. We hypothesize how mechanisms of cortical plasticity are expressed in congenitally in comparison to adventitiously blind and short-term visually deprived sighted participants and discuss potential approaches for further investigation of these mechanisms in future research.

## 1. Introduction

A key question regarding the specificity of the visual system in humans is the involvement of visual cortical areas for processing information across the senses. This review focuses on recent advances from studies conducted with both blind and sighted participants using imaging [4,6,10,12,21,30,34,35,42,43,47,50–53], transcranial magnetic stimulation (TMS) [2,5,7,11,22, 26,54,55], and electrophysiological research [1,16–20, 24,25,37–39,41,49]. Pascual-Leone and Hamilton [32] suggested that spatial processing is an implicit function of the visual system, which utilizes spatial information to perform pattern and discriminatory analyses on incoming information from other modalities, such as audition and somatosensation. For instance, a study by Haenny et al. [14] using single-cell recordings in monkeys showed clearly enhanced activity of neurons in area V4 in response to the orientation of a tactile grating. This finding was corroborated in humans by Zangaladze et al. [55], reporting that transcranial magnetic stimulation (TMS) of the middle occipital region caused a disruption in participants' abilities to discriminate orientation but not texture of a tactile grating. Furthermore, TMS and neuroimaging studies in individuals with blindsight (caused by lesions to the primary visual cortex) highlight the importance of subcortical pathways, including corticotectal and geniculostriate pathways, for conveying information to and from the visual system [3,36,46]. Reviews by Sathian [45], and Théoret, Merabet, and Pascual-Leone [48] clarified and elaborated on the rapidity of cortical plasticity following short-term visual deprivation, such as in late-blind participants and even blindfolded sighted participants performing a variety of visual and auditory tasks. However, the superior performance in a number of these tasks by early and congenitally blind individuals reinforces the necessity for understanding the mechanisms underlying both transient and robust neural plastic changes in visual cortical and extrastriate regions.

ISSN 0953-4180/08/$17.00 © 2008 – IOS Press and the authors. All rights reserved

## 2. Vision and the blind

Studies involving blind individuals have revealed clues as to how the visual cortex develops in the absence of vision. Visual cortical pathways are recruited differentially not only in sighted as compared to blind individuals but in early- as compared to late- blind individuals, suggesting that the extent of plastic reorganization varies across these populations. The primary methods implemented by researchers studying visual cortical activation in the blind are imaging and electrophysiological techniques including positron emission tomography (PET), event-related potentials (ERPs), functional magnetic resonance imaging (fMRI), and TMS. These techniques provide converging evidence of functional differences of visual cortical regions between early blind, late blind, and sighted individuals. This review discusses the findings highlighted by each technique for visual-cortical involvement in auditory and somatosensory processing in the blind.

## 3. PET

According to early PET studies (e.g., [50,52]), visual cortical activity in the blind is functionally non-specific, as evidenced by higher levels of metabolic activity in the visual cortices of early blind individuals even when they are not performing a specific task. However, recent advanced analysis techniques developed for PET research have allowed for a more fine-grained analysis of metabolic activity in the cortex of the blind [6,12, 35,43,51,53].

During performance of sound localization tasks, blind and sighted participants showed superior temporal gyrus (STG) activation which was significantly correlated with a posterior parietal lobule reference region usually active in sound localization [53]. Furthermore, for blind participants activation found in the right posterior parietal reference region was also correlated with increased activation in the dorsal visual cortical pathway. The authors suggested that there is plastic reorganization of function in congenitally blind individuals and that extrastriate occipital areas had become part of the functional network for auditory localization in their blind participants.

In a PET study where participants performed monaural and binaural sound localization tasks of noise bursts, increased cerebral blood flow (CBF) in right striate and ventral extrastriate areas was measured for a sub-group of early blind participants who performed at superior levels (the EBSP sub-group) during the monaural task in comparison to sighted control participants and a second sub-group of early blind participants with normal performance (the EBNP sub-group) [12]. In a second study which included a sample of late-blind participants, a regression analysis further indicated that the later the blind participants lost their sight, the more activation was found in the medial occipitotemporal gyrus [51]. The results of these studies point to individual differences in the extent of cortical reorganization across early blind individuals and to a role of the visual cortex particularly in processing spectral cues, which are strongly utilized during monaural sound localization [12,51]. The authors suggested that when onset of blindess occurs later, plastic reorganization of the cortex is not thorough enough to allow for increased efficiency in the performance of certain tasks, such as the monaural source discrimination task [51].

Sadato et al. [44] performed PET imaging on blind participants proficient in Braille reading to investigate tactile processing of stimuli by visual regions in the blind. All tactile discrimination tasks performed by blind but not sighted participants resulted in primary visual cortical activation [44] in comparison to non-discrimination tasks. Sadato et al. concluded that the primary visual cortex of blind individuals can reorganize in order to functionally process non-visual sensorimotor information required for task performance.

In a PET study of somatosensory activation of the visual cortex in the blind, Ptito, Moesgaard, Gjedde, and Kupers [35] used a tongue display unit (TDU) to present an electrotactile tumbling ‘T’ to congenitally blind and sighted control participants by stimulating the tongue in patterns following the position of the T on a display monitor. After training, increases in rCBF were found in the superior parietal cortex for sighted controls and in occipital cortex for blind participants. Thus, rapid plastic changes were observed in occipital regions of the congenitally blind in response to somatosensory stimulation. Cohen et al. [6] examined whether patterns of occipital involvement in somatosensory processing would be found in studies of late blind individuals. The authors did find PET activation of the left precuneus in late blind participants. Because the left precuneus is thought to be involved in visual imagery and memory recall [6], Cohen et al. posited that late blind participants may be using visual imagery to assist them in task performance.

## 4. ERPs

A successful methodological approach to investigating visual co-processing of auditory and tactile stimuli is to record multichannel ERP data in a variety of paradigms. Because traditionally ERP recordings have lacked the fine, spatial imaging abilities of fMRI and PET, they have not been as implemented in examining the role of the visual cortex in processing cross-sensory information. Localization of ERP source generators has some serious limitations. A limitless number of source configurations can potentially result in any given observed voltage distribution on the scalp (i.e., the "inverse" problem). Because of this, it is difficult to interpret specific source generators from observed scalp topography. However, ERP methodology has a temporal resolution in the millisecond range and, therefore, allows for fine-grained chronometric evaluation of the time-course of cognitive task-relevant operations. This makes it an excellent technique to consider in conjunction with fMRI, PET, or TMS. While other reviews of visual-cortical function in the blind discuss ERP findings on this area briefly, there are few which discuss this research in depth. This review discusses ERP findings regarding visual cortical involvement in auditory and tactile processing by the blind in detail to aid in creating a complete picture of not only the active regions but the time-course of visual-cortical and extrastriate processing of auditory and tactile input.

### *4.1. N1*

Early sensory processing of an auditory stimulus is indexed by a negative deflection at approximately 100–170 ms post-stimulus delivery. Earlier N1 latencies indicate greater processing efficiency of stimuli [21,38]. Röder et al. [38] investigated task-dependent activation changes both for auditory and somatosensory discrimination tasks in congenitally blind, late blind, and sighted control participants. They found that the N1 latency for blind participants was shorter, suggesting that both auditory and somatosensory information may be processed more efficiently by blind individuals.

Röder, Teder-Sälejärvi, Rösler, Hillyard, and Neville [40] further tested this hypothesis by examining the amplitude of the N1 component in sighted and blind participants in response to an auditory tuning task. Behaviorally, the results of the study showed that blind individuals were equal or better at sound localization in the periphery than sighted individuals, and the ERP data corroborated these behavioral findings. While the N1 amplitude decreased as a function of distance from the attended location, the N1 gradient was steeper to central versus peripheral stimuli in general. Notably, blind participants had a much steeper gradient to stimuli occurring in the periphery (see also [9]). This indicates that blind participants have an advantage in auditory localization specifically regarding sources to which sighted individuals are less accurate. Furthermore, the scalp distribution of the N1 was shifted to more posterior regions for blind individuals than sighted participants. Röder et al. [40] concluded that plastic changes of the cortex of blind individuals allow for compensatory processing of auditory information and that occipital regions in blind individuals are being recruited for increased processing efficiency in localizing peripheral sources.

According to Röder et al. [40], the reduction of ERP amplitude at a deflection's recovery indicates faster synapse recovery (which they refer to as the relative refractory rate) leading to greater processing efficiency. In order to test this decreasing refractoriness in auditory ERPs of blind individuals, Röder, Rösler, and Neville [39] presented congenitally blind and sighted participants with an auditory oddball task. Findings indicated that the N1 component showed a larger and faster reduction in amplitude, what the authors interpreted as synaptic recovery or refractory rate, for blind participants at temporal and parieto-occipital recording sites.

In a study by Leclerc, Saint-Amour, Lavoie, Lassonde, and Lepore [24], participants localized 30 ms broad-band noise bursts coming from one of four speakers. Blind participants performed the task more accurately and showed an N1 over occipital sites, whereas the N1 of sighted participants did not shift in topography. Leclerc et al. argued that the superior accuracy of blind participants during auditory discrimination tasks was due to more efficient stimulus feature analysis. The posterior topography of the N1 suggested that visual cortical areas were being recruited to perform this analysis.

### *4.2. Mismatch negativity* (*MMN*) *and N2 components*

Past research has shown that "deviant" infrequent tones embedded in a sequence of "frequent" standard tones usually elicit a negative deflection in the auditory ERP over frontocentral sites, emerging at approximately 150–250 ms post-stimulus onset, with an occasional right-hemispheric preponderance [27,28]. This deflection, termed mismatch negativity (MMN), is best shown as a difference wave, is unaffected by attention, and implicates early processes reflecting a short-term memory trace of preceding repetitive auditory events [28]. The MMN is frequently followed by another negative deflection, termed N2b, which is reliably observed when further processing and stimulus classification is necessary to solidify proper target identification in an attended input stream [29]. More general discrimination and classification processes of a stimulus are indexed by a negative deflection occurring at approximately 200 ms post-stimulus (termed N2). The N2 indexes half-automatic categorization mechanisms and elaborates further processing of stimulus features [8,39].

Kujala, Alho, Paavilainen, Summala, and Näätänen [20] presented blind and sighted participants with an auditory oddball location detection task in order to examine changes in scalp distribution in the MMN and N2b. For both groups the MMN was largest at fronto-central scalp sites. However, the N2b wave had a more posterior distribution in blind participants.

In a study by Alho, Kujala, Paavilainen, Summala, and Näätänen [1], using an oddball sound detection task, the topographical distribution of the MMN was more posterior for blind than for sighted participants. However, according to the authors these differences appear to be due to an overlapping N2b. This may explain the contradiction in results between this study and the study by Kujala et al. [20]. It is likely that the apparent change in the distribution of the MMN in the blind in this study was in fact due to an overlapping N2b component that occurred at posterior scalp sites in the blind. Alho et al. [1] therefore concluded that blind participants may recruit occipital and parietal brain areas in processing auditory information when the stimuli are consciously processed.

A study by Kujala et al. [17] found corroborating evidence for this hypothesis. They found more posterior N2 activation in blind participants when attending to either auditory or somatosensory deviants during a task wherein both modalities were presented to participants. This central finding of the study led the authors to conclude that visual areas in the blind appear to participate in sensory processing of auditory *and* somatosensory deviance detection when attention is necessary for task performance.

Two other studies found differential ERP activity in the occipital lobe of blind individuals. In a study by Röder et al. [39], the N2 deflection in the early blind had a higher amplitude with a more posterior topography than in sighted participants. These results suggest that blind participants may be implementing early classification mechanisms for multisensory processing and recruiting visual cortical areas to enhance processing efficiency. Kujala et al. [16] investigated the differences in auditory ERP topography between late blind, early blind, and sighted participants. They found that the N2 in late blind participants, as with early blind, showed a significant topographical shift to occipital electrode sites.

The summarized results indicate a specific role for occipital regions in the early classification of incoming auditory and somatosensory stimuli in the blind. Notably these areas were recruited when attention was required for task performance and not for the processing of unattended stimuli. The N2 and N2b, indexes of half-automatic categorization of stimuli, were more posterior in blind individuals. However, the MMN, which is fully automatic and occurs independent of attention, did not shift in topography for blind individuals while performing these tasks.

### *4.3. Slow negative potential* (*SNP*)

When participants are exposed to demanding stimulus presentation scenarios, an extended negative deflection, known as a slow negative potential, is measured from approximately 200 to 400 ms post-stimulus onset. Liotti, Ryder, and Woldorff [25] had sighted and congenitally blind participants perform an auditory oddball target discrimination task. They observed a slow negative processing component that overlapped and extended beyond the MMN and was seen in sighted and blind participants in frontal areas but with additional posterior activation in the blind [25]. Liotti et al. theorized that the left-hemispheric, posterior distribution of the slow negative potential may indicate that this region, usually involved in rapid analysis of local or high spatial frequency information, is now utilized for this same type of processing of non-visual sensory information by blind individuals.

Röder et al. [38] found a slow negative potential with a fronto-central maximum in response to auditory and somatosensory oddball conditions in blind and sighted participants that also extended over occipital cortices of blind participants. Röder et al. noted that this slow negative potential was affected by task difficulty and unaffected by stimulus modality. In a subsequent study of haptic rotation, Röder, Rösler, and Hennighausen [37] found a similar slow negativity in blind and sighted participants, which extended occipitally only in the blind. Since this slow negativity also appeared to be modality unspecific, the authors suggested it was a general co-activation of the occipital cortex during high-level cognitive processing and also low-level sensory discrimination. Further, a distinct N2 deflection seen over posterior brain areas in the blind was suggested to be an extension of this slow negative potential. The authors suggested visual cortical co-activation occurred in the blind during attention-demanding tasks, such that when frontal cortical areas were highly activated for task performance, so were occipital regions.

### *4.4. P3*

The P3 is a robust index of selective attention that varies with deviant probability and appears to reflect target recognition and updating of working memory [33]. Röder et al. [38] suggested that the P3 positive deflection is indicative of inhibitory processes involved in resetting working memory after an unexpected event. These authors found a smaller P3 amplitude shifted to anterior sites in blind individuals in response to auditory and somatosensory deviants. They suggested that the amplitude decrement at posterior sites indicated less cortical inhibition in occipital areas of the blind, meaning the occipital co-activation is not task-specific in nature but may contribute to overall processing efficiency of other modality-specific areas. In corroboration with this hypothesis, earlier studies by Kujala et al. [20] and Alho et al. [1] found that the P3 elicited in blind participants had a more anterior distribution than for the sighted as well.

In contrast, LeClerc et al. [24] reported that blind participants showed a larger P3 amplitude over parietal and occipital regions. These authors hypothesized that the large amplitude of the P3 over occipital regions in the blind suggests that blind participants recruit more attentional resources in performing cognitive tasks than the sighted. These results were corroborated by findings by Kujala et al. [16] who found a topography shift in the P3 to occipital sites in early and late blind participants.

The mixed results regarding the topography and amplitude modulation of the P3 in the blind may be due to differences in task demands across studies. The P3 indexes a wide range of cognitive processes and varies across task requirements. For example, the study by Röder et al. [38] involved a complicated oddball task that varied in difficulty, was performed across two modalities, and had four different versions. Perhaps for more complex tasks such as these, an inhibition deficit in the blind is revealed by the smaller posterior amplitude of the P3. However, for tasks requiring basic sensory or spatial discrimination and deviance detection such as the one employed by LeClerc et al. [24], the larger and more posterior P3 found in blind participants may be indicative of the recruitment of available attentional resources in occipital regions, as suggested by the authors.

## 5. fMRI

fMRI is an excellent technique for examining fine-grained regional cortical activation during task performance. This makes it an indispensible tool for studying cortical plasticity and visual cortical recruitment of function in blind individuals. Poirier, Collignon, Scheiber, and DeVolder [34] used fMRI to investigate potential occipital cortical activation during auditory motion perception in the blind. For blind participants during auditory motion processing, the left visual association area was activated in addition to the left superior lobule and premotor cortices active in sighted participants performing the same task. Poirier et al. concluded that while the superior parietal cortex and dorsal premotor cortex are involved in auditory motion processing in the blind and sighted, blind individuals also appear to recruit the occipital cortex.

In another fMRI study investigating auditory processing in blind, early blind and sighted participants were given a series of tasks where they either attended to an auditory stream with no changes in the stimulus or to an auditory stream where occasional stimulus changes occurred in the selectively attended sound sequences [21]. Occipital activation in the blind occurred only when there were occasional stimulus changes in the attended auditory stream.

Stevens, Snodgrass, Schwartz, and Weaver [47] hypothesized that selective attention mechanisms in visual regions of sighted individuals become functionally capable of acting on the reorganized cross-sensory pathways in the blind. The authors presented sighted and early blind participants with an auditory cuing task designed to elicit attention-related responses in much the same way as visual cuing paradigms except via audition. They predicted that cues indicating a discrimination trial would elicit a blood-oxygen-level dependent (BOLD) response in visual cortical areas of the early blind. Results suggested that this was indeed the case: early blind participants showed an increased preparatory BOLD response in the right medial occipital lobe. This occurred only when participants were presented with a cue predicting a discrimination trial in comparison to a cue predicting no upcoming discrimination trial. In contrast, sighted control participants showed little differences in the BOLD responses between the auditory cues. The selective attention response observed in the blind was correlated with performance, as early blind participants' peak BOLD responses in the anterior medial occipital area during the discrimination trial condition were higher the better participants performed on the task. Thus, authors concluded, the mechanisms of visual attention are functionally advantageous to early blind individuals in performing a variety of sensory tasks.

In a study of the role of the frontal eye fields during an endogenous attention auditory cuing task, Garg, Schwartz, and Stevens [10] found that while congenitally blind participants had similar and enhanced frontal eye field (FEF) activity during the delivery of auditory cues, they showed decreased activity in the medial occipital cortex. However, when the auditory target was presented following the cue, there was a sharp increase of BOLD activity in this region. The authors proposed that the medial occipital area in the congenitally blind is recruited for the processing of auditory spatial targets but not during attentional orienting. This apparently contradicts the results of Stevens et al. [47], who concluded that preparatory activation in the visual cortex during a cue was positively correlated with selective attention. However, the tasks for these two studies were quite different. While Garg et al. [10] used a spatial cuing task based on the classic Posner covert auditory attention switching paradigm, Stevens et al. used a non-spatial tone-order discrimination task, with the cue merely indicating *whether* attention would need to be engaged, not *where* it would need to be focused. The differences in task requirements may explain the apparent contradictions in findings and further suggest that occipital regions in blind participants are functionally specific, showing different patterns of activation dependent upon task demands.

Sadato et al. [43,44] confirmed that there is somatosensory activation of occipital regions in early and congenitally blind participants as well as auditory activation. These authors performed an fMRI study involving both early and late blind participants performing passive tactile tasks that either required or did not require discrimination of Braille dots [42]. Early blind participants showed increased activity in V1 in comparison to late blind participants.

Sadato et al. [42] suggested that there is a critical period of reorganization for V1 to be involved in tactile discrimination. They found that all early blind participants showed increased activity in the primary visual cortex during the tactile discrimination task while late blind and sighted participants showed decreased activity in V1. Sadato et al. [42] concluded that the visual association cortex is recruited in all blind participants in the processing of tactile shape discrimination. Additionally, in early-blind participants, primary visual cortex is involved in tasks requiring top-down processing, such as shape discrimination.

In order to examine somatosensory activation of the visual cortex during haptic discrimination by the blind without the language confound introduced by a Braille dots task, Burton, Sinclair, and McLaren [4] presented early blind, late blind, and sighted participants with a simple vibrotactile matching task while they underwent fMRI scanning. Results showed the greatest activation of visual cortical areas in early blind participants, with some visual cortical activation in several of the late blind participants. However, response magnitudes of early blind participants were greater in this area, suggesting decreased plasticity in visual cortical regions the later the onset age of blindness.

An advanced imaging technique for examining the cortical connections of neuronal fibers within and between brain regions using an MRI scanner is diffusion tensor imaging (DTI) [30]. This technique allows the researcher to analyze not only the quality of the neural connections but also the direction. Park et al. [30] performed diffusion tensor imaging on early blind and sighted control participants, specifically analyzing diffusion direction in the visual cortex. Results showed wide directional differences of white matter tracts between blind and sighted participants arriving and leaving the visual cortex and other structures related to visual processing, including the superior longitudinal fasciculus and the inferior cerebellar peduncle. This supports evidence of at least partial functional rewiring in the visual cortices of early blind participants.

## 6. TMS

TMS is a technique where localized magnetic pulses are applied to the surface of the scalp and can either activate or inhibit specific underlying cortical areas depending on the frequency of stimulation (e.g., [23,54]). At more rapid, repeated stimulation rates, it is known as repetitive TMS (rTMS). It allows for the examination of specific regional cortical participation in task performance and is often used in conjunction with fMRI

or PET scanning (e.g., [54]). Cohen et al. [5] implemented TMS to examine somatosensory processes that would be disrupted during stimulation to visual cortical regions in early blind versus sighted individuals. When TMS was applied over occipital positions, the blind participants in a Braille reading condition reported that dots seemed to be missing, that extra phantom dots appeared, or that the dot pattern did not make sense. Fewer errors were made by blind participants when they were stimulated over their somatosensory cortices and parietal sites, while sighted participants made many more errors during sensorimotor stimulation and very few during occipital stimulation. Cohen et al. hypothesized that occipital cortical regions in blind individuals, but not sighted, are functionally involved in somatosensory processing and that there may be generalized occipital activation for early blind participants performing attentional tasks (such as auditory and somatosensory discrimination tasks). Although as of yet the results of this study elude replication, they were corroborated in a case-study by Hamilton, Keenan, Catala, and Pascual-Leone [15]. In this study a woman blind from birth developed alexia for Braille after a stroke causing extensive bilateral lesions in her occipital lobe. The results of these two studies [5,15] suggest that the occipital cortex is at least partially engaged in decoding spatial and tactile information necessary for Braille reading.

Kupers, Fumal, de Noordhout, Gjedde, Schoenen, and Ptito [22] applied TMS to early blind, late blind, and sighted participants before and after training on the TDU. TMS was applied to occipital cortical regions and primary somatosensory cortex (S1). Before training, TMS stimulation of occipital regions caused several blind participants to feel sensations on their fingertips. After training, TMS of the occipital cortex caused three of the eight early blind participants, and one of the late blind participants, to feel sensations on the tongue. Sensations were not elicited after stimulation of S1. Sighted participants only reported the induction of phosphenes, and not tongue sensations, during occipital stimulation. The authors suggested that there was already cross-sensory tactile rerouting to the visual cortex in their blind participants but that these pathways were initially shaped from input from the fingers and expanded to tongue input after training with the TDU. These same authors found that rTMS stimulation of the middle occipital cortex in early blind participants, but not in sighted controls, abolished repetition priming effects during Braille reading [23]. They suggested these findings give support to the role of the occipital cortex in implicit memory formation in the early blind.

In order to asses changes in visual cortical excitability over a range of loss of vision, Gothe, Brandt, Irlbacher, Röricht, Sabel, and Meyer [11] applied TMS to the occipital lobe of three groups of blind participants with differing amounts of residual vision and a group of sighted control participants. The authors hypothesized that stimulation would cause visual phosphenes depending on the level of residual visual cortical function across participant groups. Phosphenes were elicited in all of the sighted control participants, in all blind participants with measurable residual vision, in 60% of blind participants with residual light and motion sensitivity, and in only 20% of blind participants with no residual vision. Thus, the less residual vision participants possessed the less TMS stimulation of their occipital cortex elicited phosphenes. Gothe et al. concluded that long-term deafferentation of the visual cortex causes reorganization such that individuals become less able to experience cortically elicited phosphenes.

Merabet et al. [26] performed rTMS stimulation on the occipital cortices of congenitally blind participants who performed roughness or spatial distance discrimination of Braille dots. Stimulation of the occipital cortex caused deficits in performance of spatial discrimination, a macrogeometric judgment, while stimulation of the somatosensory cortex preferentially disrupted roughness discriminations, a microgeometric judgment. Further support was collected through testing a Braille alexic patient who had lost her ability to read Braille following a bilateral occipital stroke [15]. Her performance on the spatial judgment task was highly impaired while she could still perform the roughness discrimination task [26]. These findings suggest that visual cortical areas may preferentially process macrogeometric information such as spatial density and can perform this task across any sensory modality.

Repetitive TMS disruption of the occipital cortex has also implicated occipital involvement in semantic processing and verb generation in the early blind [2]. Repetitive TMS stimulation of the left lateral occipital cortex and the left posterior calcarine fissure (V1) leads to high semantic errors in a verb generation task in comparison to sham or S1 stimulation. Semantic errors in sighted participants were elicited, in turn, by stimulation of the left inferior prefrontal cortex, the area classically involved in language processing in non-blind populations. These findings suggest that early retinotopic regions traditionally involved in visual analysis become recruited in a more extensive network for performing high-level cognitive tasks.

Another study provided support for involvement by the dorsal visual pathway in auditory localization by early blind participants [7]. Early blind and sighted participants were trained in the use of a visual prosthesis unit (PSVA) which acts as a substitute for vision by delivering auditory information via differences in pitch, intensity, and spatial location. Rapid TMS of right dorsal extrastriate in early blind participants only interfered specifically with their ability to utilize spatial location information while using the PSVA. Collignon et al. [7] suggested this further implicates the dorsal pathway's involvement in spatial analysis of cross-sensory information in the blind, perhaps via the unmasking or extension of neural pathways devoted to the processing of non-visual spatial information (e.g., [31]).

Wittenberg, Werhahn, Wassermann, Herscovitch, and Cohen [54] implemented rTMS to stimulate (as opposed to disrupt) S1 areas in the early blind. This led to increased regional CBF in visual areas of the early blind which was not found in late blind or sighted control participants. The authors hypothesized a strengthening of already existing links between parietal and occipital regions in the early blind.

## 7. Potential mechanisms for plastic reorganization

Pascual-Leone and Hamilton [32] discussed possible mechanisms for plastic reorganization in the brain, with a focus on visual cortical activation to tactile and haptic stimulation in the blind, as measured with PET, fMRI, and TMS. Blind-folded, sighted participants were deprived of vision for 5 days. During this five day period, participants performed tactile and auditory matching tasks while undergoing fMRI. Results showed successive recruitment of occipital cortex during task performance and a decrease in somatosensory cortical activation during the tactile task. On the last day of the experiment, participants also underwent rTMS to the occipital cortex. This rTMS stimulation significantly disrupted the participants' ability to discriminate Braille symbols tactilely. Within a day following removal of the blindfold, fMRI no longer revealed occipital activation during auditory and tactile tasks, and rTMS to the occipital cortex no longer disrupted tactile discrimination. The authors hypothesized that even in sighted populations the visual cortex receives visual, auditory, and tactile input, and subserves spatial discrimination tasks in all three sensory modalities. Once visual deafferentation occurs, as in the case of individuals who are born or become blind, the extent of auditory and tactile connections are more widely revealed.

A recent impressive review by Pascual-Leone, Amedi, Fregni, and Merabet [31] also discussed potential mechanisms for plastic change in the visual cortex. This detailed review discussed plastic reorganization of the visual cortex in blind individuals in the processing of haptic, tactile, and advanced cognitive and verbal information. After comparing short-term plastic changes in the sighted when temporarily deprived of vision and long-term plastic changes in the congenitally blind, the authors proposed a two-stage account of neural plasticity during visual deprivation. They reasoned that when the visual cortex is deprived of visual input, established somatosensory and auditory connections to the visual cortex are 'unmasked' in stage one, and in stage two new connections are established, leading to a more widespread and generalized plastic reorganization of the cortex over time.

In support of the two-stage theory, Merabet et al. [26] found BOLD activation of primary visual cortex in sighted participants performing tactile discrimination tasks. Participants were blind-folded for only 90 minutes before these activation patterns were observed. Further, suppression of extrastriate cortical areas was observed simultaneously. The authors proposed a dual-source hypothesis to explain these results. They proposed that, in fully functioning sighted individuals, hierarchical feedback pathways which operate through the dorsal stream cause inhibition of extrastriate areas during performance of a non-visual task. Meanwhile, competing, direct neural pathways from other sensory modalities cause co-activation of primary visual areas during non-visual task performance. This theory is partially corroborated by studies of mental chronometry revealed through ERPs. Results of smaller amplitudes in the parieto-occipital P3 in blind individuals [20,38] suggest the proposed dorsal stream feedback pathways are not as developed or efficient in blind participants. This is to be expected since there is no need for inhibition of visual information when non-visual processing must occur.

## 8. Conclusion and discussion

There is convincing evidence that visual cortical and extrastriate regions are functionally involved in processing non-visual sensory information in populations who have lost their vision both early and later in life. This suggests plastic reorganization of neural pathways or the existence of already formed connections which are revealed once a visual deficit occurs. Differences in the extent of visual cortical recruitment in early blind, late blind, and visually deprived sighted populations suggest that a variety of such mechanisms exist.

PET studies of hemodynamic activation [6,12,35,43, 51,53] have suggested visual cortical areas are recruited for auditory and somatosensory task performance in the blind, specifically during auditory localization, monaural localization and discrimination (which require a heavy dependence on spectral cue information), and somatosensory discrimination.

While ERP methodology has a limitation in that it does not allow for fine spatial localization of function, it has an advantage in that it allows for fine-grained temporal analysis of the chronometry of mental processing stages. In this sense, ERPs complement the arsenal of research tools by virtue of their temporal resolution in the millisecond range. The evidence provided by ERPs suggests more efficient processing of peripheral auditory source cues by blind participants in comparison to sighted as implicated by earlier latencies and steeper gradients of the auditory N1 [38,40,41]. Further, recruitment of visual areas in the blind occurs at stages of processing when discrimination and categorization takes place, as implicated by the posterior topographies of the N2 and N2b during auditory and tactile discrimination tasks (e.g., [17,16,38]). There has been no shift observed in the MMN [1,20], an index of early automatic short-term memory processes independent of attention [28]. This suggests that recruitment of visual cortex in the blind affects stages of processing which occur after both initial sensory gating and initial automatic short-term memory trace formation.

The P3 is an intriguing candidate for showing differences in congenitally blind populations exposed to different task requirements. One theory states that the P3 indicates a state of widespread inhibition that resets the contents of working memory for more efficient processing of new information [13]. Kujala et al. [20] and Röder et al. [37,38] found a shift in the P3 to more anterior sites in the blind. These authors [37,38] hypothesized that an inhibition deficit in the blind may be the reason for a decrease in the amplitude of the posterior P3. This proposition was supported by findings of a smaller posterior P3 amplitude in blind individuals during a complex study of somatosensory and auditory perceptual discrimination [38] and was related specifically to task relevance and probability during a study of haptic transformation [37]. However, Kujala et al. [16] and LeClerc et al. [24] found a more posterior shift of the P3 in blind compared to sighted participants. One hypothesis for these differences is that for the less demanding oddball discrimination tasks presented by Kujala et al. [16] and Leclerc et al. [24], an enhanced occipital P3 indexes recruitment of available attentional resources through occipital neural sources, whereas tasks with a higher processing load tax the available resources of the blind and reveal deficits in the inhibition mechanism indexed by the P3.

Functionally, fMRI studies have implicated the involvement of visual cortical and extrastriate regions during non-visual task performance in blind as compared to sighted participants. BOLD activation of visual cortical association areas was observed during auditory motion processing by blind participants [34]. In corroboration with ERP research, increased BOLD activation in occipital regions of blind participants was observed specifically when attention was necessary for task performance and not when the stimuli were ignored or unnoticed [10,21,47]. There is also fMRI evidence for visual cortical activation during performance of tactile discrimination tasks by the early blind [42] in agreement with PET imaging studies [6,43] and ERP studies [17,38,42]. Furthermore, a DTI study indicated that intact white matter tracts were observed arriving and leaving the visual cortex of early blind participants which differed in nature from those of sighted participants [30]. This suggests plastic rewiring of an active network of functional connections in the visual cortices of early blind participants.

TMS, a relatively new tool which allows for discreet disruption and stimulation of cortex and is excellent for studying cortical functional relevance and cortical networks of function, has also revealed potential mechanisms of cortical plasticity in the blind. TMS studies indicated disruption of tactile spatial discrimination specifically during occipital stimulation in blind participants [5,23,26,44]. Together, this body of research has revealed the involvement and activation of visual cortex in the processing of non-visual information, particularly if that information is high-acuity spatial information actively attended in either the auditory or somatosensory modality. Functional MRI and modern imaging methodology have allowed for clear and specific examination of the precise regional contributions of visual cortex and extrastriate regions during the performance of sensory and cognitive operations by blind and sighted individuals. Combined with TMS methodology, which allows for the brief and localized stimulation or inhibition of discreet areas of cortex, we can begin to create an accurate picture of where and what cortical regions are contributing to non-visual sensory processing in the blind. Due to their temporal resolution in the millisecond range, ERP recordings are ideally suited to accurately delineate whether plasticity as a mechanism is indeed characterized by a sequence of distinct, conditional processes.

An expanded examination of the two-stage hypothesis presented by Pascual-Leone et al. [31] is necessary to elaborate on a mechanism as to how both a first, short-term 'unmasking' stage and a second, long-term plastic reorganization stage are expressed and achieved. ERP studies of early and late blind individuals reveal topographical shifts in early discrimination waveforms such as the N2. In accordance with these findings, short-term visual deafferentation in the occipital cortex may lead to the revelation of somatosensory and auditory inputs that allow for precise, spatial discrimination processes to be performed by the visual cortex as proposed by Pascual-Leone and Hamilton [32]. One way this hypothesis can be further tested is by implementing ERPs to probe potential shifts in the N2 in normally sighted participants who have been deprived of vision for a short period of time (such as via blindfolding). It can be predicted that a posterior topographical shift of the N2 will be seen, since several sighted participants showed occipital BOLD activation during an auditory discrimination study by Kujala et al. [21]. Differences in P3 findings in the early blind across studies may also reveal answers as to how more long-term plastic reorganization is being expressed after prolonged visual deprivation. During attention-demanding or complex tasks, this more long-term deprivation may lead to a general recruitment of occipital regions that is subserved by a lack of cortical inhibition mechanisms in these regions.

## References

[1] K. Alho, T. Kujala, P. Paavilainen, H. Summala and R. Näätänen, Auditory processing in visual brain areas of the early blind: Evidence from event-related potentials, *Electroencephalographic Clinical Neurophysiology* **86** (1993), 418–487.

[2] A. Amedi, A. Floel, S. Knecht, E. Zohary and L.G. Cohen, Transcranial magnetic stimulation of the occipital pole interferes with verbal processing in blind subjects, *Nature Neuroscience* **7** (2004), 1266–1270.

[3] J.L. Boyer, S. Harrison and T. Ro, Unconscious processing of orientation and color without primary visual cortex, *Proceedings of the National Academy of Sciences USA* **102** (2005), 16875–16879.

[4] H. Burton, R.J. Sinclair and D.G. McLaren, Cortical activity to vibrotactile stimulation: An fMRI study in blind and sighted individuals, *Human Brain Mapping* **23** (2004), 210–228.

[5] L.G. Cohen, P. Celnik, A. Pascual-Leone, B. Corwell, L. Faiz, J. Dambrosia, M. Honda, N. Sadato, C. Gerloff, M.D. Catalá and M. Hallett, Functional relevance of cross-modal plasticity in blind humans. *Nature* **389** (1997), 180–183.

[6] L.G. Cohen, R.A. Weeks, N. Sadato, P. Celnik, K. Ishii and M. Hallett, Period of susceptibility for cross-modal plasticity in the blind, *Annals of Neurology* **45** (1999), 451–460.

[7] O. Collignon, M. Lassonde, F. Lepore, D. Bastien and C. Veraart, Functional Cerebral Reorganization for Auditory Spatial Processing and Auditory Substitution of Vision in Early Blind Subjects, *Cerebral Cortex* **17** (2007), 457–465.

[8] M. Eimer, Mechanisms of visuospatial attention: Evidence from event-related brain potentials, *Visual Cognition* **5** (1998), 257–286.

[9] A. Fieger, B. Röder, W.A. Teder-Sälejärvi, S.A. Hillyard and H. Neville, Auditory spatial tuning in late-onset blindness in humans, *Journal of Cognitive Neuroscience* **18** (2006), 149–157.

[10] A. Garg, D. Schwartz and A.A. Stevens, Orienting auditory spatial attention engages frontal eye fields and medial occipital cortex in congenitally blind humans, *Neuropsychologia* **45** (2007), 2307–2321.

[11] J. Gothe, S.A. Brandt, K. Irlbacher, S. Röricht, B.A. Sabel and B.U. Meyer, Changes in visual cortex excitability in blind subjects as demonstrated by transcranial magnetic stimulation, *Brain* **125** (2002), 479–490.

[12] F. Gougoux, R.J. Zatorre, M. Lassonde, P. Voss and F.A. Lepore, Functional neuroimaging study of sound localization: Visual cortex activity predicts performance in early-blind individuals, *PLoS Biology* **3** (2005), 0324–0333.

[13] S. Grossberg, Processing of expected and unexpected events during conditioning and attention: a psychophysiological theory, *Psychological Review* **889** (1982), 529–572.

[14] P.E. Haenny, J.H. Maunsell and P.H. Schiller, State dependent activity in monkey visual cortex I. Single cell activity in V1 and V4 on visual tasks, *Experimental Brain Research* **69** (1988), 245–59.

[15] R. Hamilton, J.P. Keenan, M. Catala and A. Pascual-Leone, Alexia for Braille following bilateral occipital stroke in an early blind woman, *Cognitive Neuroscience* **11** (2000), 237–240.

[16] T. Kujala, K. Alho, M. Huotilainen, R.J. Ilmoniemi, A. Lehtokoski, A. Leinonen, T. Rinne, O. Salonen, J. Sinkkonen, C.J. Standertskjöld-Nordenstam and R. Näätänen, Electrophysiological evidence for cross-modal plasticity in humans with early- and late-onset blindness, *Psychophysiology* **34** (1997), 213–216.

[17] T. Kujala, M. Huotilainen, J. Sinkkonen, A. Ahonen, K. Alho, M. Hämäläinen, R. Ilmoniemi, M. Kajola, J. Knuutila, J. Lavikainen, O. Salonen, J. Simola, C. Standertskjöld-Nordenstam, H. Tiitinen, S. Tissari and R. Näätänen, Visual cortex activation in blind humans during sound discrimination, *Neuroscience Letters* **183** (1995), 143–146.

[18] T. Kujala, K. Alho, J. Kekoni, H. Hämäläinen, K. Reinikainen, O. Salonen, C.-G. Standertskjöld-Nordenstam and R. Näätänen, Auditory and somatosensory event-related brain potentials in early blind humans, *Experimental Brain Research* **104** (1995), 519–526.

[19] T. Kujala, K. Alho and R. Näätänen, Cross-modal reorganization of human cortical functions, *Trends in Neuroscience* **23** (2000), 115–120.

[20] T. Kujala, K. Alho, P. Paavilainen, H. Summala and R. Näätänen, Neural plasticity in processing of sound location by the early blind: An event-related potential study, *Elec-*

*troenephalography and Clinical Neurophysiology: Evoked Potentials* **84** (1992), 469–472.

[21] T. Kujala, M.J. Palva, O. Salonen, P. Alku, M. Huotilainen, Järvinene and R. Näätänen, The role of blind humans' visual cortex in auditory change detection, *Neuroscience Letters* **379** (2005), 127–131.

[22] R. Kupers, A. Fumal, A.M. de Noordhout, A. Gjedde, J. Schoenen and M. Ptito, Transcranial magnetic stimulation of the visual cortex induces somatotopically organized qualia in blind subjects, *Proceedings of the National Academy of Sciences USA* **103** (2006), 13256–13260.

[23] R. Kupers, M. Pappens, A.M. de Noordhout, J. Schoenen, M. Ptito and A. Fumal, rTMS of the occipital cortex abolishes Braille reading and repetition priming in blind subjects, *Neurology* **68** (2007), 691–693.

[24] C. Leclerc, D. Saint-Amour, M. Lavoie, M. Lassonde and F. Lepore, Brain Functional reorganization in early blind humans revealed by auditory event-related potentials, *Neuroreport* **11** (2000), 545–550.

[25] M. Liotti, K. Ryder and M.G. Woldorff, Auditory attention in the congenitally blind: Where, when and what gets reorganized? *Neuroreport* **9** (1998), 1007–1012.

[26] L. Merabet, G. Thut, B. Murray, J. Andrews, S. Hsiao and A. Pascual-Leone, Feeling by sight or seeing by touch? *Neuron* **42** (2004), 173–179.

[27] R. Näätänen, Mismatch negativity (MMN): Perspectives for application, *International Journal of Psychophysiology* **37** (2000), 3–10.

[28] R. Näätänen, P. Paavilainen, T. Rinne and K. Alho, The mismatch negativity (MMN) in basic research of central auditory processing: a review, *Clinical Neurophysiology* **118** (2007), 2544–2590.

[29] R. Näätänen and T. Picton, The N1 wave of the human electric and magnetic response to sound: A review and an analysis of the component structure, *Psychophysiology* **24** (1987), 375–425.

[30] H.J. Park, S.O. Jeong, E.Y. Kim, J.I. Kim, H. Park, M.K. Oh, D.J. Kim, S.Y. Kim, S.C. Lee and J.D. Lee, Reorganization of neural circuits in the blind on diffusion direction analysis, *Neuroreport* **18** (2007), 1757–1760.

[31] A. Pascual-Leone, A. Amedi, F. Fregni and L.B. Merabet, The plastic human cortex, *Annual Reviews of Neuroscience* **28** (2005), 377–401.

[32] A. Pascual-Leone and R. Hamilton, The metamodal organization of the brain, in: *Progress in Brain Research*, (Vol. 134), Casanova and Ptito, eds, 2001, pp. 1-19.

[33] T.W. Picton, The P300 wave of the human event-related potential, *Journal of Clinical Neurophysiology* **9** (1992), 456–479.

[34] C. Poirier, O. Collignon, C. Scheiber and A. De Volder, Auditory motion processing in early blind subjects, *Cognitive Processing* **5** (2004), 254–256.

[35] M. Ptito, S.M. Moesgaard, A. Gjedde and R. Kupers, Cross-modal plasticity revealed by electrotactile stimulation of the tongue in the congenitally blind, *Brain* **128** (2005), 606–614.

[36] T. Ro and R. Rafal, Visual restoration in cortical blindness: insights from natural and TMS-induced blindsight, *Neuropsychological Rehabilitation* **16** (2006), 377–396.

[37] B. Röder, F. Rösler and E. Hennighausen, Different cortical activation patterns in blind and sighted humans during encoding and transformation of haptic images, *Psychophysiology* **34** (1997), 292–307.

[38] B. Röder, F. Rösler, E. Hennighausen and F. Näcker, Event-related potentials during auditory and somatosensory discrimination in sighted and blind human subjects, *Cognitive Brain Research* **4** (1996), 77–93.

[39] B. Röder, F. Rösler and H.J. Neville, Effects of interstimulus interval on auditory event-related potentials in congenitally blind and normally sighted humans, *Neuroscience Letters* **264** (1999), 53–56.

[40] B. Röder, W.A. Teder-Sälejärvi, F. Rösler, S.A. Hillyard and H. Neville, Improved auditory spatial tuning in blind humans, *Nature* **400** (1999), 162–166.

[41] C. Rorden and J. Driver, Does auditory attention shift in the direction of an upcoming saccade? *Neuropsychologia* **37** (1999), 357–377.

[42] N. Sadato, T. Okada, M. Honda and Y. Yonekura, Critical period for cross- modal plasticity in blind humans: a functional MRI study, *NeuroImage* **16** (2002), 389–400.

[43] N. Sadato, A. Pascual-Leone, J. Grafman, M.P. Deiber, V. Ibanex and M. Hallett, Neural networks for Braille reading by the blind, *Brain* **121** (1999), 1213–1229.

[44] N. Sadato, A. Pascual-Leone, J. Grafman, V. Ibañez, M.P. Deiber, G. Dold and M. Hallett, Activation of the primary visual cortex by Braille reading in blind subjects, *Nature* **380** (1996), 526–528.

[45] K. Sathian, Visual cortical activity during tactile perception in the sighted and the visually deprived, *Developmental Psychobiology* **46** (2005), 279–286.

[46] J. Silvanto, A. Cowey, N. Lavie and V. Walsh, Making the blindsighted see, *Neuropsychologia* **45** (2007), 3346–3350.

[47] A.A. Stevens, M. Snodgrass, D. Schwartz and K. Weaver, Preparatory activity in occipital cortex in early blind humans predicts auditory perceptual performance, *Journal of Neuroscience* **27** (2007), 10734–10741.

[48] H. Théoret, L. Merabet and A. Pascual-Leone, Behavioral and neuroplastic changes in the blind: evidence for functionally relevant cross-modal interactions, *Journal of Physiology-Paris* **98** (2004), 221–233.

[49] F. Uhl, P. Franzen, G. Lindinger, W. Lang and L. Deecke, On the functionality of the visually deprived occipital cortex in early blind persons, *Neuroscience Letters* **124** (1991), 256–259.

[50] C. Veraart, A.G. DeVolder, M.C. Wanet-Defalque, A. Bol, C. Michel and A.M. Goffinet, Glucose utilization in human visual cortex is abnormally elevated in blindness of early onset but decreased in blindness of late onset, *Brain Research* **510** (1990), 115–121.

[51] P. Voss, F. Gougoux, R.J. Zatorre, M. Lassonde and F. Lepore, Differential occipital responses in early- and late-blind individuals during a sound-source discrimination task, *Neuroimage* **40** (2008), 746–758.

[52] M.C. Wanet-Defalque, C. Veraart, A. DeVolder and R. Metz, C. Michel, G. Dooms and A. Goffinet, High metabolic activity in the visual cortex of early blind human subjects, *Brain Research* **446** (1988), 369–373.

[53] R. Weeks, B. Horwitz, A. Aziz-Sultan, B. Tian, C.M. Wessinger, L.G. Cohen, M. Hallett and J.P. Rauschecker, A positron emission tomographic study of auditory localization in the congenitally blind, *Journal of Neuroscience* **20** (2000), 2664–2672.

[54] G.F. Wittenberg, K.J. Werhahn, E.M. Wassermann, P. Herscovitch and L.G. Cohen, Functional connectivity between somatosensory and visual cortex in early blind humans, *European Journal of Neuroscience* **20** (2004), 1923–1927.

[55] A. Zangaladze, C.M. Epstein, S.T. Grafton and K. Sathian, Involvement of visual cortex in tactile discrimination of orientation, *Nature* **401** (1999), 587–590.
